# Unicornuate Uterus and Rudimentary Horn: An Unusual Cause of Recurrent Fetal Malpresentation Identified During a Scheduled Cesarean Delivery

**DOI:** 10.7759/cureus.46277

**Published:** 2023-09-30

**Authors:** Shaikh Muneeba, Neema Acharya, Shazia Mohammad, Lucky S Reddy, Aishwarya Gupta

**Affiliations:** 1 Obstetrics and Gynecology, Jawaharlal Nehru Medical College, Datta Meghe Institute of Higher Education and Research, Wardha, IND

**Keywords:** multidisciplinary collaboration, obstetric anomaly, cesarean delivery, recurrent fetal malpresentation, rudimentary horn, unicornuate uterus

## Abstract

This case report presents a unique clinical scenario involving a 32-year-old woman with a unicornuate uterus and a rudimentary horn, leading to recurrent fetal malpresentation. The patient, gravida 3, para 1, presented at 36 weeks of gestational age with contractions and vaginal bleeding. Clinical evaluation revealed a breech presentation and nonreassuring fetal heart tracings. An emergency cesarean section was performed, during which a unicornuate uterus with a rudimentary horn was identified and fused to the main uterine cavity on the left posterior aspect. The rudimentary horn bore a functional left tube and ovary. The surgical procedure was successful, resulting in the delivery of a healthy baby girl. This case underscores the importance of meticulous preoperative assessment, multidisciplinary collaboration, and informed consent in managing complex uterine anomalies to ensure optimal maternal and fetal outcomes.

## Introduction

Uterine anomalies constitute a diverse spectrum of congenital malformations that can profoundly influence the course of pregnancy. Among these, the unicornuate uterus with a rudimentary horn is a rare and intriguing anomaly that presents unique challenges in obstetric management [1]. This case report delves into the clinical narrative of a 32-year-old woman with a unicornuate uterus and a rudimentary horn, the manifestation of which led to recurrent fetal malpresentation. By illuminating this distinctive clinical scenario, we aim to shed light on the intricacies of diagnosing and managing such an anomaly, emphasizing the importance of thorough assessment and interdisciplinary collaboration for successful outcomes in cases of complex uterine malformations [2].

Uterine anomalies, though infrequent, encompass a diverse range of structural deviations from the normal uterine anatomy. These anomalies can be classified into various categories, such as fusion defects, septal malformations, and agenesis, each with its distinct impact on reproductive outcomes [3]. The unicornuate uterus, characterized by a single uterine horn, poses unique challenges due to its asymmetry and potential association with a rudimentary contralateral horn. The rudimentary horn, often underdeveloped and nonfunctional, can harbor various obstetric complications, making timely recognition and proper management essential for optimal maternal and fetal outcomes [4].

Recurrent fetal malpresentation, a common consequence of uterine anomalies, can complicate pregnancy and childbirth. Malpresentation refers to the deviation of the fetal presenting part from the vertex position, increasing the risks of prolonged labor, obstructed delivery, and potential birth trauma. In this context, the unicornuate uterus with a rudimentary horn introduces an additional layer of complexity to the understanding and management of recurrent fetal malpresentation [5].

This case report presents a comprehensive analysis of the clinical course of a patient with a unicornuate uterus and a rudimentary horn, where the anomaly played a pivotal role in the recurrent presentation of fetal malposition. This case serves as a testament to the importance of understanding and addressing uterine malformations in obstetric practice and underscores the significance of sharing such clinical experiences to enrich the medical community's knowledge and expertise in managing challenging cases.

## Case presentation

The patient, a 32-year-old woman, gravida 3, para 1, had a history of one previous cesarean section. She had been married for seven years and had a three-year-old male child delivered through cesarean section due to breech presentation. Additionally, she experienced one spontaneous abortion at eight weeks of gestational age. Her medical history was unremarkable, with no history of gestational hypertension, diabetes mellitus, or other associated comorbidities.

**Figure 1:**
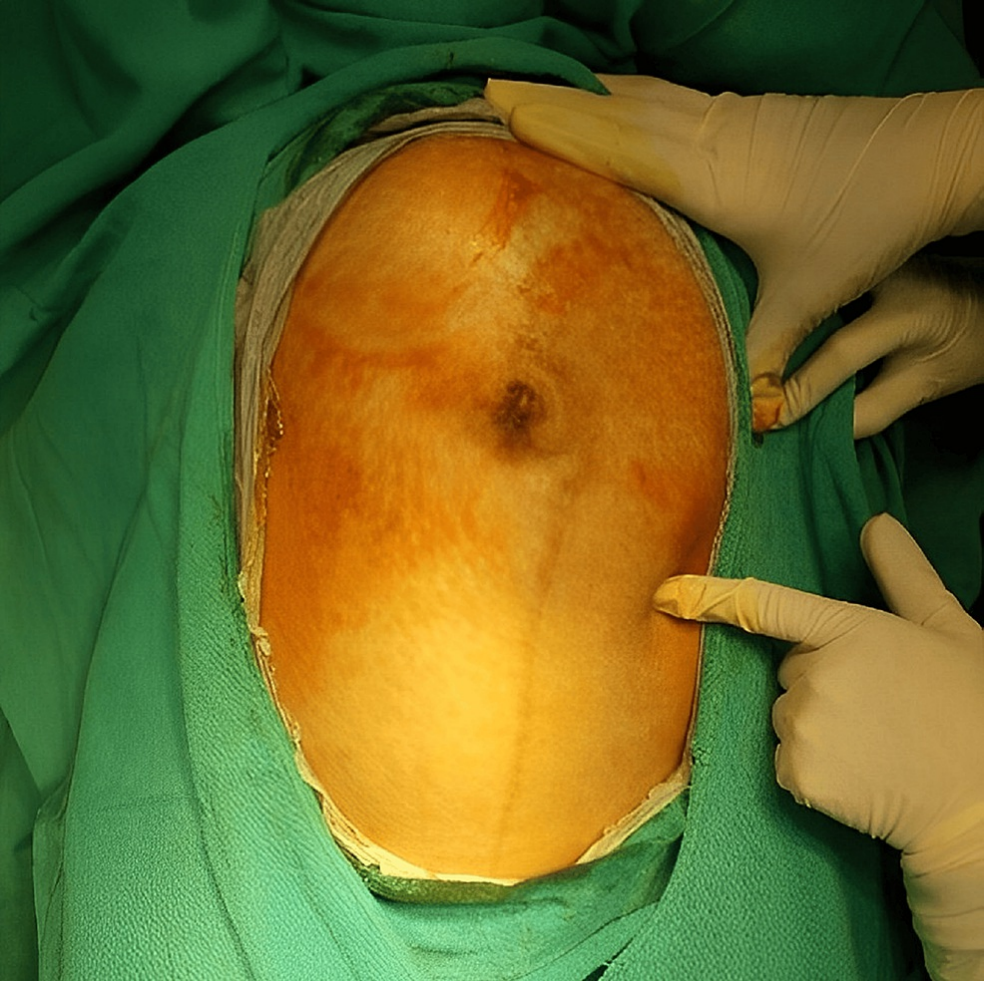
Identification of a longitudinal fetal lie, where the fetal head is situated at the fundal pole. At the same time, the buttocks are located at the podalic end, indicating a consistent breech presentation.

At 36 weeks of gestational age, the patient presented to the emergency department complaining of contractions and slight vaginal bleeding. On physical examination, several pertinent findings were noted. The linea nigra and striae gravidarum were visible on the abdominal skin. A previous cesarean section scar was evident. Fetal movements were visible upon inspection. The fundal height of the uterus reached the xiphisternum, indicating appropriate uterine growth. A longitudinal fetal lie was detected, with the fetal head positioned at the fundal pole and the buttocks at the podalic end, consistent with breech presentation (Figure 1). Fetal heart sounds were auscultated at a rate of 148 bpm, exhibiting regular rhythm. Cardiotocography (CTG) indicated a nonreassuring nonstress test (NST) with variable decelerations, slight reactivity, and beat-to-beat variability.

The patient's most recent ultrasound scan revealed a single live intrauterine fetus at 36+3 weeks of estimated gestational age. The estimated fetal weight was 2.5 kg. The breech presentation was confirmed, and the placenta was situated anteriorly with adequate amniotic fluid levels. The thickness of the cesarean scar was measured at 3.5 mm.

Given the clinical presentation and the findings from antenatal imaging, the patient was promptly prepared for an emergency cesarean section. Informed consent was obtained, and the patient was informed of the risks associated with the surgical procedure. These risks included those related to the cesarean section, the possibility of needing obstetric hysterectomy, potential placenta accreta, the requirement for blood transfusion, NICU admission for the newborn, and the option for tubal ligation during the surgery.

The medical team involved in the patient's care included the attending obstetrician, a pediatrician, and an anesthetist. The pediatrician was informed about the case to ensure prompt assessment and care of the newborn. At the same time, the anesthetist was briefed about the patient's medical history and the potential need for specialized care during anesthesia administration.

Under spinal anesthesia, a Pfannenstiel incision was made to access the uterus. The surgical team delivered a baby girl weighing 2.3 kg. Apgar scores were recorded at 8 and 9 at one and five minutes, respectively. The cesarean section was performed successfully and without complications.

**Figure 2:**
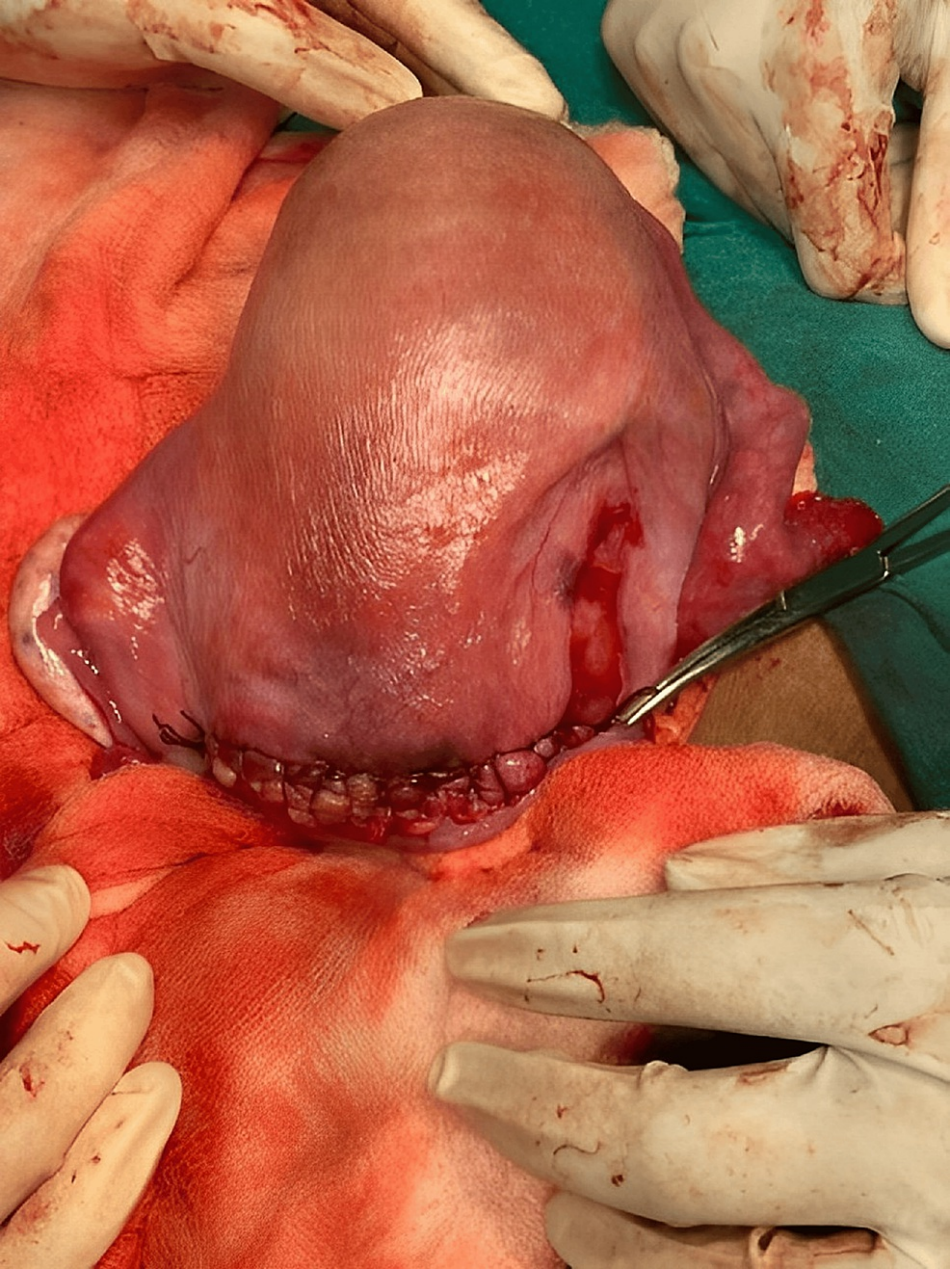
Visualization of a unicornuate uterus with an accompanying rudimentary horn. The rudimentary horn, situated on the right side of the unicornuate uterus, was observed to be flattened in its appearance.

During the surgery, a thorough examination of the uterus revealed a unicornuate uterus with a rudimentary horn. The rudimentary horn was flattened and located on the right side of the unicornuate uterus (Figure 2). It was fused to the main uterine cavity on the left posterior side. The left tube and ovary were attached to the rudimentary horn, adding complexity to the anatomical arrangement. The patient's postoperative course was uneventful, and routine clinical follow-up was conducted for 48 hours after the surgery. The patient and the newborn were discharged in stable condition.

## Discussion

The presented case underscores the complex nature of congenital uterine anomalies and their significant implications for pregnancy outcomes. In particular, the unicornuate uterus with a rudimentary horn presents intricate challenges that demand thorough understanding and careful management. Congenital uterine anomalies arise from anomalies in the development of Müllerian ducts during fetal life. These anomalies exhibit varying prevalence rates across different populations. While affecting 1-10% of the general population, the prevalence rises to 2-8% in infertile women and 5-30% in those with a history of miscarriages [6,7]. The presence of maternal uterine anomalies amplifies the risk of diverse obstetric complications, including preterm birth, preterm premature rupture of membranes, breech presentation, cesarean section, placenta previa, placental abruption, and intrauterine growth retardation (IUGR) [8]. These findings highlight the need for heightened vigilance and tailored management in women with uterine anomalies to optimize pregnancy outcomes.

The rarity of a unicornuate uterus, found in only 0.1% of the general population, presents unique challenges. Women with this anomaly experience poor reproductive outcomes, including a live birth rate of merely 29.2%, a high prematurity rate of 44%, and an ectopic pregnancy rate of 4% [9]. Additionally, the risk of first and second-trimester abortions and intrauterine fetal demise is notably elevated [10]. The compromised reproductive performance is attributed to abnormal uterine blood flow, decreased muscle mass in the unicornuate uterus, and cervical incompetence, all contributing to adverse pregnancy outcomes.

In the case presented, a rudimentary horn in conjunction with a unicornuate uterus introduces further complexities. The rudimentary horn is often non-communicating, and its occurrence is associated with ectopic pregnancies. The unique mechanism of pregnancy occurrence within a non-communicating rudimentary horn through transperineal migration of sperm or fertilized ovum is rare, with an estimated frequency of approximately 1 in 76,000 pregnancies. However, this anomaly poses a substantial risk of uterine rupture, necessitating vigilant monitoring [11].

This case report underscores the paramount significance of meticulous preoperative assessment and multidisciplinary collaboration in managing complex uterine anomalies. Successful surgical intervention hinges upon careful navigation through intricate anatomy. In this case, the patient's sufficient blood supply to the dominant uterine cavity and the possible presence of normal endometrium within it likely contributed to her successful full-term pregnancy without significant fetal compromise.

## Conclusions

The presented case vividly illustrates the complexities and challenges associated with uterine anomalies in obstetric management, specifically the unicornuate uterus with a rudimentary horn. The recurrent fetal malpresentation, in this case, underscores the crucial role that uterine anatomy plays in shaping pregnancy outcomes. By meticulously recounting the patient's journey from presentation to surgical intervention, we have highlighted the importance of thorough preoperative assessment, multidisciplinary collaboration, and informed decision-making. Managing uterine anomalies requires a comprehensive approach integrating obstetric, surgical, anesthetic, and neonatal considerations. The identification of a unicornuate uterus and its associated rudimentary horn during a scheduled cesarean delivery showcases the necessity for obstetricians, gynecologists, pediatricians, and anesthetists to work in tandem to achieve favorable outcomes for both the mother and the newborn. This case report underscores the significance of vigilant antenatal care and imaging in early anomaly detection, allowing for tailored management strategies. The successful cesarean delivery with careful surgical navigation and attention to the unique uterine anatomy is a testament to the importance of surgical precision and patient-centered care in rare uterine malformations.
